# Poor glycaemic control is associated with increased risk of neurodevelopmental disorders in childhood-onset type 1 diabetes: a population-based cohort study

**DOI:** 10.1007/s00125-020-05372-5

**Published:** 2021-01-16

**Authors:** Shengxin Liu, Ralf Kuja-Halkola, Henrik Larsson, Paul Lichtenstein, Jonas F. Ludvigsson, Ann-Marie Svensson, Soffia Gudbjörnsdottir, Magnus Tideman, Eva Serlachius, Agnieszka Butwicka

**Affiliations:** 1 Department of Medical Epidemiology and Biostatistics, Karolinska Institutet, Solna, Sweden; 2 School of Medical Sciences, Örebro University, Örebro, Sweden; 3 Department of Pediatrics, Örebro University Hospital, Örebro, Sweden; 4 Division of Epidemiology and Public Health, School of Medicine, University of Nottingham, Nottingham, UK; 5 Department of Medicine, Columbia University College of Physicians and Surgeons, New York, NY USA; 6 Swedish National Diabetes Register, Centre of Registers, Gothenburg, Sweden; 7 Department of Molecular and Clinical Medicine, Sahlgrenska Academy, University of Gothenburg, Gothenburg, Sweden; 8 School of Health and Welfare, Halmstad University, Halmstad, Sweden; 9 Child and Adolescent Psychiatry, Stockholm Health Care Service, Region Stockholm, Sweden; 10 Department of Child Psychiatry, Medical University of Warsaw, Warsaw, Poland

**Keywords:** Attention-deficit/hyperactivity disorder, Autism spectrum disorders, Glycaemic control, Intellectual disability, Neurodevelopmental disorders, Type 1 diabetes

## Abstract

**Aims/hypothesis:**

The aim of this study was to investigate the effect of childhood-onset type 1 diabetes on the risk of subsequent neurodevelopmental disorders, and the role of glycaemic control in this association. We hypothesised that individuals with poor glycaemic control may be at a higher risk of neurodevelopmental disorders compared with the general population, as well as compared with individuals with type 1 diabetes with adequate glycaemic control.

**Methods:**

This Swedish population-based cohort study was conducted using data from health registers from 1973 to 2013. We identified 8430 patients with childhood-onset type 1 diabetes (diagnosed before age 18 years) with a median age of diabetes onset of 9.6 (IQR 5.9–12.9) and 84,300 reference individuals from the general population, matched for sex, birth year and birth county. Cox models were used to estimate the effect of HbA_1c_ on the risk of subsequent neurodevelopmental disorders, including attention-deficit/hyperactivity disorder (ADHD), autism spectrum disorders (ASD) and intellectual disability.

**Results:**

During a median follow-up period of 5.6 years, 398 (4.7%) individuals with type 1 diabetes received a diagnosis of any neurodevelopmental disorder compared with 3066 (3.6%) in the general population, corresponding to an adjusted HR (HR_adjusted_) of 1.31 (95% CI 1.18, 1.46) after additionally adjusting for other psychiatric morbidity prior to inclusion, parental psychiatric morbidity and parental highest education level. The risk of any neurodevelopmental disorder increased with HbA_1c_ levels and the highest risk was observed in patients with mean HbA_1c_ >8.6% (>70 mmol/mol) (HR_adjusted_ 1.90 [95% CI 1.51, 2.37]) compared with reference individuals without type 1 diabetes. In addition, when compared with patients with diabetes with HbA_1c_ <7.5% (<58 mmol/mol), patients with HbA_1c_ >8.6% (>70 mmol/mol) had the highest risk of any neurodevelopmental disorder (HR_adjusted_ 3.71 [95% CI 2.75, 5.02]) and of specific neurodevelopmental disorders including ADHD (HR_adjusted_ 4.16 [95% CI 2.92, 5.94]), ASD (HR_adjusted_ 2.84 [95% CI 1.52, 5.28]) and intellectual disability (HR_adjusted_ 3.93 [95% CI 1.38, 11.22]).

**Conclusions/interpretation:**

Childhood-onset type 1 diabetes is associated with an increased risk of neurodevelopmental disorders, with the highest risk seen in individuals with poor glycaemic control. Routine neurodevelopmental follow-up visits should be considered in type 1 diabetes, especially in patients with poor glycaemic control.

**Graphical abstract:**

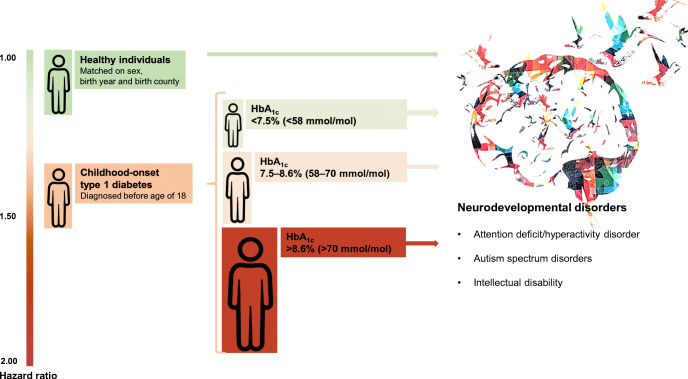

**Supplementary Information:**

The online version contains peer-reviewed but unedited supplementary material available at 10.1007/s00125-020-05372-5.

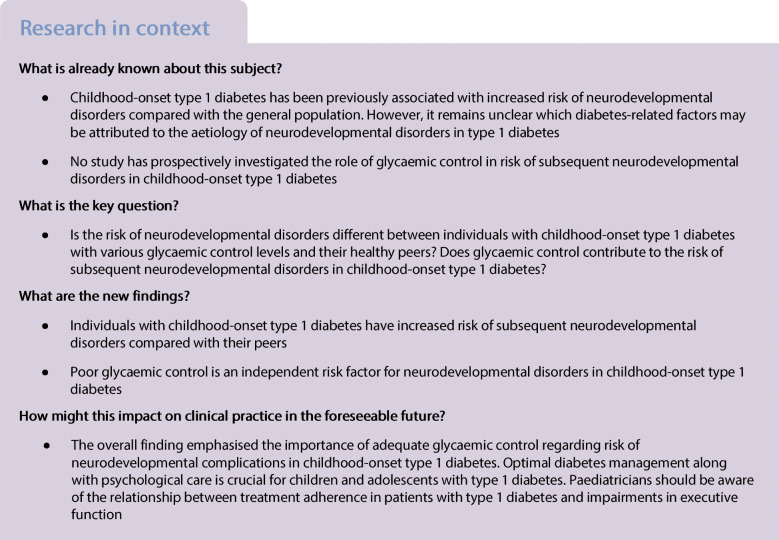



## Introduction

Type 1 diabetes is the predominant form of diabetes in childhood and is increasing worldwide [1]. Several studies have suggested a link between childhood-onset type 1 diabetes and increased risk of neurodevelopmental disorders, including attention-deficit/hyperactivity disorder (ADHD), autism spectrum disorders (ASD) and intellectual disability [2–4].

Neurodevelopmental disorders are a group of conditions classified together by the Diagnostic and Statistical Manual of Mental Disorders (5th edition) [5] because of their common onset during childhood, high comorbidity rate of 20–80% and essential overlap of contributing factors across specific diagnoses [6].

Although the aetiology of neurodevelopmental disorders is often attributed to genetic factors in the general population [7, 8], a different biological mechanism may be more plausible in type 1 diabetes. In our previous study, statistically significant increased risk of neurodevelopmental disorders (HRs 1.5–1.7) was observed in individuals with childhood-onset type 1 diabetes, but not in their healthy siblings. This suggests that diabetes-related factors play important roles in the aetiology of neurodevelopmental disorders in individuals with diabetes rather than shared genetic and environmental influences [2].

Glucose metabolism is essential for brain development and function [9], and its disturbance during childhood may have negative consequences. Adverse effects of dysglycaemic insults on central nervous system development in children and adolescents with type 1 diabetes have long been recognised [10–12]. Several studies have shown that poor glycaemic control is related to impairments in neurocognitive functions, such as attention (the ability to focus selectively on certain information and sustain that focus while disregarding other perceivable stimuli), working memory (capacity to temporarily store information in order to execute cognitive tasks) and intellectual abilities [13–15]. Yet, it remains unknown whether these neurocognitive deficits can be translated into increased risk of medical diagnosis of neurodevelopmental disorders or merely indicate subclinical difficulties.

Many children and adolescents in high-income countries fail to achieve the recommended target of HbA_1c_ [16]. Diabetes guidelines emphasise the importance of appropriate glycaemic control to avoid microvascular and microvascular complications [17]. Nevertheless, there is limited evidence regarding whether maintaining adequate glycaemic control similarly benefits paediatric patients through reduced psychological morbidity risk. A better understanding of the relationship between glycaemic control and the risk of neurodevelopmental disorders is also crucial for future evidence-based recommendations on neurodevelopmental follow-ups, and early interventions. A recent Danish population-based study found that poor glycaemic control with an average HbA_1c_ level of >8.6% (>70 mmol/mol) predicted a higher risk of psychiatric morbidity in individuals with type 1 diabetes [18], but this study was unable to specifically address the risk of neurodevelopmental disorders, which are aetiologically distinct from other psychiatric disorders [19].

In this study, we used Swedish registers, which have high coverage and contain prospectively collected information on HbA_1c_ measurements and medical diagnoses, to investigate the effect of childhood-onset type 1 diabetes on the subsequent risk of neurodevelopmental disorders, and the role of glycaemic control in this association.

## Methods

### Study design

For this population-based cohort study, consistent with previous studies [2, 20], we used data from nationwide registers in Sweden to compose a cohort of patients with type 1 diabetes and a matched reference cohort. Detailed descriptions of these registers are presented in electronic supplementary material (ESM) Table 1. In brief, we used the Swediabkids database and the Swedish Diabetes Register to identify patients born in Sweden from 1973 onwards with childhood-onset type 1 diabetes diagnosed before age 18 years. We only included individuals with HbA_1c_ measurement within 1 year after diabetes diagnosis. For each patient, we randomly selected ten individuals for comparison, matched on sex, birth year and birth county, from the Total Population Register. Individuals with chromosomal abnormalities and individuals diagnosed with neurodevelopmental disorders prior to inclusion (date of first registration for patients and the corresponding date for matched reference individuals) were excluded.

Patients with type 1 diabetes and matched reference individuals without type 1 diabetes were followed from the inclusion date to the date of first diagnosis of any neurodevelopmental disorder, emigration, death or end of follow-up (31 December 2013), whichever came first. Information on date of death or emigration was extracted from the Cause of Death Register or the Migration Register.

### HbA_1c_

HbA_1c_ was reported according to the International Federation of Clinical Chemistry standard in mmol/mol and converted into percentage according to the Diabetes Control and Complication Trial [21]. Accuracy of HbA_1c_ measurement in involved care units was ensured by an external quality assessment scheme [22]. Mean HbA_1c_ was calculated as AUC divided by the time interval between the first and last recorded HbA_1c_. AUC was estimated using the trapezoidal method, which accounted for time intervals between HbA_1c_ measurements. For consistency with previous research [18], HbA_1c_ levels were categorised as <7.5% (<58 mmol/mol), 7.5–8.6% (58–70 mmol/mol), and >8.6% (>70 mmol/mol). Matched reference individuals served as reference glycaemic control levels with presumed normal HbA_1c_, a method that has been used in previous research [20].

### Neurodevelopmental disorders

Neurodevelopmental disorders were identified from four registries: the National Patient Register, the Clinical databases for Child and Adolescent Mental Health Services, the Habilitation Register and the Halmstad University Register on Pupils with Intellectual Disability in accordance with ICD codes (ESM Table 2). Similar to previous research in paediatric psychiatry [23, 24], we decided to study neurodevelopmental disorders as a group of conditions. Our primary outcome was any neurodevelopmental disorder and secondary outcomes were specific neurodevelopmental disorders including: (1) ADHD; (2) ASD; and (3) intellectual disability.

### Covariates

Other psychiatric morbidity was defined as at least one diagnosis of the following prior to the inclusion date: anxiety, mood disorders, psychotic disorders, eating disorders, psychoactive substance misuse or other behavioural disorder (corresponding ICD codes presented in ESM Table 2). Information on patients’ episodes of severe hypoglycaemia (defined as unconsciousness or seizures) and diabetic ketoacidosis was obtained from Swediabkids from 2008 onwards [25]. Information on biological parents’ characteristics was obtained by linking to the Multi-Generation Register. Parental psychiatric morbidity was defined as any psychiatric diagnosis, including diagnosis of any neurodevelopmental disorders and other psychiatric disorders mentioned above, prior to the inclusion date of the child. Data on parental highest education level were acquired from the Education Register, the longitudinal integration database for health insurance and labour market studies, and the population censuses from the years of 1970, 1975 and 1985.

### Statistical analyses

Baseline characteristics were calculated across patients with different mean HbA_1c_ levels and matched reference individuals, and presented as means (SD) or median (IQR) for continuous variables, or percentage for categorical variables.

#### Comparison with the general population

We used Cox models to estimate HRs with 95% CIs for all outcomes between type 1 diabetes patients with different mean HbA_1c_ levels and matched reference individuals. First, we fitted crude models where patients with diabetes were compared with their matched reference individuals (matched on sex, birth year and birth county) using a Cox model stratified with each matched group entered as a stratum. Then, we fitted models additionally adjusted for other psychiatric morbidity prior to inclusion, parental psychiatric morbidity and parental highest education level to calculate adjusted HRs (HR_adjusted_).

#### Comparison among patients using time-varying HbA_1c_

We also used Cox models to evaluate the association between different HbA_1c_ levels and outcomes among individuals with type 1 diabetes. We modelled HbA_1c_ levels as time-varying variables, where the follow-up time was split each time when the HbA_1c_ level changed. For instance, if a patient was in category >8.6% (>70 mmol/mol), the patient contributed person-years to the >8.6% (>70 mmol/mol) category. If the same patient switched to 7.5–8.6% (58–70 mmol/mol), the patient then added person-years to the 7.5–8.6% (58–70 mmol/mol) category. HbA_1c_ level was evaluated as a categorical variable and as a continuous variable for each 1% (10 mmol/mol) change. Model 1 was adjusted for time-varying diabetes duration using a Cox model stratified on age at diabetes diagnosis and birth year. Model 2 was additionally adjusted for sex, other psychiatric morbidity prior to inclusion, parental psychiatric morbidity and parental highest education level. Model 3 was additionally adjusted for time-varying occurrence of severe hypoglycaemia and diabetic ketoacidosis in patients diagnosed after 2008, as this information was collected after then.

#### Sensitivity analyses

The analyses were repeated in a subsample of individuals with type 1 diabetes excluding those diagnosed with any neurodevelopmental disorders within 1 year after diabetes diagnosis to minimise the risk of undiagnosed existing neurodevelopmental disorders and/or detection bias. The analyses were also repeated in patients whose first documented HbA_1c_ was within the first 3 months after diagnosis. We additionally repeated the analyses by categorising HbA_1c_ according to glycaemic targets proposed by the International Society for Pediatric and Adolescent Diabetes (ISPAD): <7.0% (<53 mmol/mol), 7.0–8.6% (53–70 mmol/mol) and >8.6% (>70 mmol/mol) [17].

Tests were two-tailed and conducted at the 0.05 significance level. All data management was conducted in SAS Software version 9.4 (SAS Institute, USA) and statistical analyses were performed using statistical software R version 3.6.1 (https://cran.r-project.org/bin/windows/base/old/3.6.1/).

### Ethics approval

This study was approved by the Regional Ethical Review Board in Stockholm (2013/862-31/5). Anonymised data were obtained from Statistics Sweden and no patients or individuals from the general population were contacted due to the register-based nature of this study.

## Results

### Baseline characteristics

**Fig. 1:**
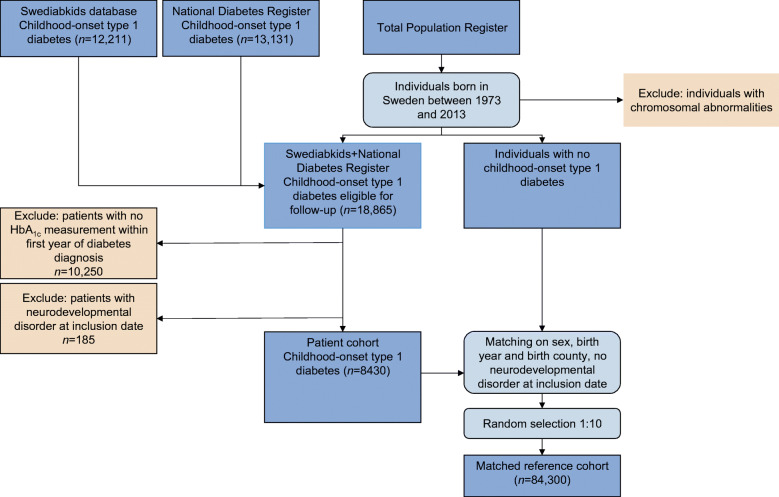
Flowchart of the patient cohort of childhood-onset type 1 diabetes (diagnosed before age of 18 years) and matched reference individuals including detailed information on the excluded individuals

We included 8430 individuals diagnosed with childhood-onset type 1 diabetes (diagnosed before age of 18 years) and 84,300 matched reference individuals from the general population (Fig. 1). As shown in Table 1, matching worked well and distributions of age at inclusion, sex and birth year were identical between patients and matched individuals. We found no statistically significant differences between patients with type 1 diabetes and matched individuals for other psychiatric morbidity prior to inclusion or parental psychiatric morbidity. The parental highest education level of patients with type 1 diabetes was slightly higher than that of matched reference individuals. For individuals with type 1 diabetes, the median baseline HbA_1c_ was 7.5 (IQR 6.6–8.7), with HbA_1c_ measured every 3.2 months (median, IQR 2.6–3.9).

**Table 1:** Baseline characteristics of individuals with childhood-onset type 1 diabetes (diagnosed before age of 18 years) and matched reference individuals from the general population

Variable	Overall type 1 diabetes	Type 1 diabetes grouped by mean HbA_1c_ during follow-up			Matched reference individuals^a^
		<7.5%	7.5–8.6%	>8.6%	
		(<58 mmol/mol)	(58–70 mmol/mol)	(>70 mmol/mol)	
Sex, *n* (%)					
Male	4571 (54.2)	2150 (55.2)	1869 (54.3)	552 (50.4)	45,710 (54.2)
Female	3859 (45.8)	1744 (44.8)	1572 (45.7)	543 (49.6)	38,590 (45.8)
Age at the start of follow-up, year					
Mean (SD)	9.5 (4.4)	9.4 (4.6)	9.0 (4.2)	11.0 (3.8)	9.5 (4.4)
Median (IQR)	9.6 (5.9–12.9)	9.4 (5.5–13.2)	9.1 (5.7–12.2)	11.2 (8.5–13.6)	9.5 (5.9–12.9)
Age at the end of follow-up, year					
Mean (SD)	14.2 (5.4)	13.8 (5.6)	16.1 (5.1)	18.9 (4.3)	15.4 (5.6)
Median (IQR)	15.7 (11.3–19.8)	13.6 (9.3–18.0)	16.5 (12.6–20.0)	19.7 (16.3–21.9)	15.7 (11.4–19.8)
Length of follow-up, year					
Mean (SD)	5.9 (3.8)	4.3 (3.4)	7.1 (3.6)	7.9 (3.8)	5.9 (3.9)
Median (IQR)	5.6 (2.7–8.7)	3.5 (1.6–6.3)	6.9 (4.4–9.6)	7.9 (5.5–10.4)	5.6 (2.7–8.7)
Reasons for the end of follow-up, *n* (%)					
Neurodevelopmental disorder	398 (4.7)	119 (3.1)	181 (5.3)	98 (9.0)	3066 (3.6)
Death	15 (0.2)	5 (0.1)	6 (0.2)	4 (0.4)	84 (0.1)
Emigration	72 (0.9)	29 (0.7)	29 (0.8)	14 (1.3)	1309 (1.6)
End of the study	7945 (94.2)	3741 (96.1)	3225 (93.7)	979 (89.4)	79,840 (94.7)
Baseline HbA_1c_, %					
Mean (SD)	7.9 (1.9)	7.6 (1.8)	8.1 (1.9)	8.4 (2.0)	
Median (IQR)	7.5 (6.6–8.7)	7.2 (6.5–8.3)	7.6 (6.9–8.8)	8.1 (7.0–9.3)	
Baseline HbA_1c_, mmol/mol					
Mean (SD)	63 (20.4)	59 (19.5)	65 (20.2)	69 (20.2)	
Median (IQR)	58 (49–72)	55 (47–67)	60 (52–73)	65 (53–78)	
Other psychiatric morbidity prior to inclusion, *n* (%)	74 (0.88)	33 (0.85)	23 (0.67)	18 (1.64)	719 (0.85)
Parental psychiatric morbidity, *n* (%)					
Paternal	598 (7.1)	264 (6.8)	235 (6.8)	99 (9.0)	6191 (7.3)
Maternal	745 (8.8)	350 (9.0)	291 (8.5)	104 (9.5)	7945 (9.4)
Parental highest education level, *n* (%)					
Primary and lower secondary education	176 (2.1)	45 (1.2)	73 (2.1)	58 (5.3)	2666 (3.2)
Upper secondary education	3715 (44.1)	1484 (38.1)	1600 (46.5)	631 (57.6)	36,428 (43.2)
Postsecondary education	4538 (53.8)	2365 (60.7)	1768 (51.4)	405 (37.0)	45,170 (53.6)
Missing	1 (0.01)	0	0	1 (0.1)	36 (0.04)

^a^Matched on sex, birth year and birth county

### Comparison with the general population

Over a median follow-up time of 5.6 years, 398 (4.7%) patients (7.95/1000 person-years) and 3066 (3.6%) matched reference individuals (6.12/1000 person-years) received at least one diagnosis of neurodevelopmental disorders. After adjustment for covariates (other psychiatric morbidity prior to inclusion, parental psychiatric morbidity and parental highest education level), individuals with childhood-onset type 1 diabetes were at statistically significantly higher risk of any neurodevelopmental disorders (HR_adjusted_ 1.31 [95% CI 1.18, 1.46]), ADHD (HR_adjusted_ 1.29 [95% CI 1.14, 1.46]) and ASD (HR_adjusted_ 1.31 [95% CI 1.04, 1.65]) compared with matched reference individuals (Table 2).

**Table 2:** Incidence rate and HRs with 95% CIs for neurodevelopmental disorders between individuals with childhood-onset type 1 diabetes (diagnosed before age of 18 years) and matched reference individuals from general population according to mean HbA_1c_ levels

Variable	Matched reference individuals	Overall type 1 diabetes	Type 1 diabetes grouped by mean HbA_1c_ during follow-up		
			<7.5% (<58 mmol/mol)	7.5–8.6% (58–70 mmol/mol)	>8.6% (>70 mmol/mol)
Any neurodevelopmental disorders					
Number of outcomes /person-years	3066/501,314	398/50,051	119/16,888	181/24,487	98/8676
Incidence rate/per 1000 person-years^a^	6.12	7.95	7.05	7.39	11.30
Crude model^b^	1 (reference)	1.29 (1.17, 1.44)	1.07 (0.89, 1.30)	1.21 (1.03, 1.41)	2.10 (1.69, 2.62)
Adjusted model^c^	1 (reference)	1.31 (1.18, 1.46)	1.15 (0.95, 1.39)	1.22 (1.05, 1.43)	1.90 (1.51, 2.37)
ADHD					
Number of outcomes /person-years	2313/501,314	297/50,051	77/16,888	142/24,487	78/8676
Incidence rate/per 1000 person-years	4.61	5.93	4.56	5.80	8.99
Crude model	1 (reference)	1.28 (1.14, 1.45)	0.93 (0.73, 1.17)	1.25 (1.05, 1.48)	2.25 (1.76, 2.88)
Adjusted model	1 (reference)	1.29 (1.14, 1.46)	0.99 (0.78, 1.25)	1.26 (1.06, 1.51)	1.98 (1.53, 2.55)
ASD					
Number of outcomes /person-years	654/501,314	84/50,051	39/16,888	32/24,487	13/8676
Incidence rate/per 1000 person-years	1.30	1.68	2.31	1.31	1.50
Crude model	1 (reference)	1.28 (1.02, 1.61)	1.45 (1.04, 2.03)	1.07 (0.74, 1.54)	1.47 (0.82, 2.64)
Adjusted model	1 (reference)	1.31 (1.04, 1.65)	1.54 (1.09, 2.16)	1.07 (0.74, 1.54)	1.48 (0.82, 2.68)
Intellectual disability					
Number of outcomes /person-years	307/501,314	40/50,051	10/16,888	20/24,487	10/8676
Incidence rate/per 1000 person-years	0.61	0.80	0.59	0.82	1.15
Crude model	1 (reference)	1.29 (0.93, 1.79)	1.02 (0.53, 1.95)	1.27 (0.79, 2.02)	1.84 (0.94, 3.63)
Adjusted model	1 (reference)	1.35 (0.96, 1.90)	1.12 (0.58, 2.18)	1.39 (0.86, 2.24)	1.61 (0.80, 3.23)

^a^Number of outcomes per 1000 person-years

^b^Crude model: Cox regression stratified on each matched group as the stratum

^c^Adjusted model: additionally adjusted for other psychiatric morbidity prior to inclusion, parental psychiatric morbidity and parental highest education level

Patients with a mean HbA_1c_ level <7.5% (<58 mmol/mol) did not differ statistically significantly from matched reference individuals regarding risk for subsequent neurodevelopmental disorders and ADHD, although gradually increased risks were observed at higher mean HbA_1c_ levels. For any neurodevelopmental disorders, adjusted HRs increased from 1.22 (95% CI 1.05, 1.43) at mean HbA_1c_ level 7.5–8.6% (58–70 mmol/mol) to 1.90 (95% CI 1.51, 2.37) at mean HbA_1c_ level >8.6% (>70 mmol/mol). For ADHD, adjusted HRs increased from 1.26 (95% CI 1.06, 1.51) at mean HbA_1c_ level 7.5–8.6% (58–70 mmol/mol) to 1.98 (95% CI 1.53, 2.55) at mean HbA_1c_ level >8.6% (>70 mmol/mol). In contrast, patients with mean HbA_1c_ level <7.5% (<58 mmol/mol) displayed a significantly higher risk of ASD (HR_adjusted_ 1.54 [95% CI 1.09, 2.16]), while the risk in patients at higher mean HbA_1c_ levels was not statistically significantly different from the matched reference individuals. The risk of intellectual disability was not statistically significantly increased in individuals regardless of their glycaemic control.

### Comparison among patients using time-varying HbA_1c_

Table 3 shows the estimates from Cox models using time-varying HbA_1c_ among patients with type 1 diabetes, where the risks of neurodevelopmental disorders gradually increased with higher HbA_1c_ levels. Compared with HbA_1c_ <7.5% (<58 mmol/mol), the adjusted HR for any neurodevelopmental disorders was 1.64 (95% CI 1.22, 2.21) for HbA_1c_ of 7.5–8.6% (58–70 mmol/mol) and increased to 3.71 (95% CI 2.75, 5.02) for HbA_1c_ >8.6% (>70 mmol/mol). Similar patterns were observed for ADHD, where HR_adjusted_ increased from 1.73 (95% CI 1.21, 2.46) to 4.16 (95% CI 2.92, 5.94). The risk of ASD and intellectual disability did not differ statistically significantly between the two lowest HbA_1c_ levels (<7.5% [<58 mmol/mol] and 7.5–8.6% [58–70 mmol/mol]), while patients with HbA_1c_ >8.6% (>70 mmol/mol) displayed a statistically significantly increased risk of both disorders with an adjusted HR of 2.84 (95% CI 1.52, 5.28) for ASD and 3.93 (95% CI 1.38, 11.22) for intellectual disability. When time-varying HbA_1c_ was analysed as a continuous variable, an increase of 1% (10 mmol/mol) was statistically significantly associated with increased risk of any neurodevelopmental disorders (HR_adjusted_ 1.38 [95% CI 1.29, 1.47]), ADHD (HR_adjusted_ 1.45 [95% CI 1.35, 1.55]) and intellectual disability (HR_adjusted_ 1.41 [95% CI 1.11, 1.79]). Because of the availability of information, additional adjustment for episodes of severe hypoglycaemia and diabetic ketoacidosis was done for patients diagnosed after 2008, and led to similar conclusions (ESM Table 3).

**Table 3:** HRs with 95% CIs for neurodevelopmental disorders among individuals with childhood-onset type 1 diabetes (diagnosed before age of 18 years) according to time-varying HbA_1c_ levels

Variable	Time-varying HbA_1c_ levels			HbA_1c_ 1% (10 mmol/mol) change
	<7.5% (<58 mmol/mol)	7.5–8.6% (58–70 mmol/mol)	>8.6% (>70 mmol/mol)	
Any neurodevelopmental disorders				
Model 1^a^	1 (reference)	1.55 (1.18, 2.04)	3.68 (2.81, 4.82)	1.37 (1.30, 1.46)
Model 2^b^	1 (reference)	1.64 (1.22, 2.21)	3.71 (2.75, 5.02)	1.38 (1.29, 1.47)
ADHD				
Model 1	1 (reference)	1.64 (1.19, 2.28)	4.21 (3.06, 5.80)	1.43 (1.34, 1.53)
Model 2	1 (reference)	1.73 (1.21, 2.46)	4.16 (2.92, 5.94)	1.45 (1.35, 1.55)
ASD				
Model 1	1 (reference)	1.36 (0.79, 2.36)	2.34 (1.35, 4.07)	1.11 (0.95, 1.30)
Model 2	1 (reference)	1.58 (0.86, 2.90)	2.84 (1.52, 5.28)	1.14 (0.97, 1.35)
Intellectual disability				
Model 1	1 (reference)	1.76 (0.76, 4.07)	3.84 (1.61, 9.18)	1.40 (1.12, 1.75)
Model 2	1 (reference)	1.95 (0.67, 5.64)	3.93 (1.38, 11.22)	1.41 (1.11, 1.79)

^a^Model 1: Cox regression stratified on age at type 1 diabetes diagnosis and birth year, adjusted for time-updated diabetes duration

^b^Model 2: additionally adjusted for sex, other psychiatric morbidity prior to inclusion, parental psychiatric morbidity and parental highest education level

Sensitivity analyses of restricting outcomes to neurodevelopmental disorders diagnosed after 1 year of diabetes diagnosis (ESM Table 4 and 5), restricting study population to patients with type 1 diabetes whose first documented HbA_1c_ was performed within the first 3 month after diagnosis (ESM Table 6 and 7) and using ISPAD HbA_1c_ cut-offs (ESM Table 8 and 9) yielded similar results.

## Discussion

In this large population-based cohort study, we found that individuals with childhood-onset type 1 diabetes were at higher risk of neurodevelopmental disorders compared with their peers from the general population and that this risk increased at higher mean HbA_1c_ levels. We also demonstrated that poor glycaemic control, which was assessed using time-varying HbA_1c_, was an independent risk factor for subsequent neurodevelopmental disorders in childhood-onset type 1 diabetes.

To date, two population-based studies have evaluated the risk of neurodevelopmental disorders in individuals with childhood-onset type 1 diabetes compared with the general population. In our previous study of 17,122 children and adolescents with type 1 diabetes, we observed 1.5–1.7-fold increased risk of ADHD, ASD and intellectual disability diagnosed before the age of 18 years [2, 26]. A Danish study of 5084 patients with childhood-onset type 1 diabetes reported slightly (statistically non-significant) increased risk of ADHD in girls (HR_adjusted_ 1.12 [95% CI 0.81, 1.56]) and ASD in boys (HR_adjusted_ 1.09 [95% CI 0.82, 1.46]) diagnosed after diabetes onset [26].

Our current study, consisting of 8430 individuals with childhood-onset type 1 diabetes and with a focus on the first event of neurodevelopmental disorders diagnosed after diabetes onset, observed statistically significantly elevated risks with adjusted HRs ranging from 1.27 to 1.30. The somewhat lower HRs observed in the current study, compared with our previous study [2], could be explained by the exclusions of patients with chromosomal abnormalities and patients with neurodevelopmental disorders prior to diabetes onset, and a focus on recent years (the current study included type 1 diabetes diagnosed 1995–2013 as opposed to 1973–2009 in our previous study). Another study from the Danish group observed an association between high mean HbA_1c_ level (>8.6% [>70 mmol/mol]) during the first 2 years after diabetes onset and later diagnosis of any psychiatric disorders. Yet, the summarised outcome ‘any psychiatric disorders’ included a wide range of diagnoses with different aetiological backgrounds, such as neurodevelopmental disorders but also depression and anxiety [18]. Our study is the first presenting specific risks of neurodevelopmental disorders in childhood-onset type 1 diabetes compared with the general population and within patients. A novel aspect of the current study is the monotonic increase in the risk of all types of neurodevelopmental disorders with higher HbA_1c_ values, which may be explained by the use of the time-varying HbA_1c_ in our analysis. This method of estimation of glycaemic control is known to be more accurate than using mathematical mean or single measurement of HbA_1c_ [27].

Our findings suggest that maintaining adequate glycaemic control is important for controlling potential psychological burdens in childhood-onset type 1 diabetes, since patients with adequate glycaemic control showed no statistically significant difference in risk of any neurodevelopmental disorders compared with the general population. Notably, risks of any neurodevelopmental disorders and ADHD gradually increased at higher glycaemic control, and nearly doubled in patients with poor glycaemic control compared with their peers without type 1 diabetes. Although in the comparison with the general population, statistically significantly increased risk of ASD was only found in patients with adequate glycaemic control, this result was not confirmed when we restricted our analysis to individuals with type 1 diabetes only, where the risk was almost tripled (HR_adjusted_ 2.84) in patients with poor glycaemic control compared with those adequately controlled. We noted that there was limited statistical power in these analyses, but we cannot exclude the possibility that some of these results were a result of patients with type 1 diabetes with high awareness of disease management more frequently and willingly seeking advice from medical professionals, and thus being more likely to be psychiatrically evaluated and receive a timely diagnosis than their peers without type 1 diabetes.

Several possible mechanisms may explain the observed increased risk of neurodevelopmental disorders in individuals with type 1 diabetes, especially in those with poor glycaemic control. Studies have found inadequate growth of grey and white matter, of both microstructure and volume, in children with type 1 diabetes, especially in those with chronic hyperglycaemia (i.e. poor glycaemic control) [28–30]. A similar pattern of hindered growth of cortical surface area and hippocampus volume has also been reported in other studies [29, 31]. On one hand, the compromised brain growth and neurodevelopment in type 1 diabetes may lead to altered neuropsychological functions, and subsequently clinically diagnosed neurodevelopmental disorders. Abnormalities in the above-mentioned brain structures are commonly present in individuals with neurodevelopmental disorders [32], for instance, hippocampus deficits are often found in individuals with ADHD and ASD, and correspond with memory and executive impairments [33]. On the other hand, it may worsen the pre-existing subthreshold symptoms of the undiagnosed neurodevelopmental disorders so they become clinically significant. Moreover, detection bias may contribute to the observed association, as individuals with type 1 diabetes need to be closely monitored and thus have increased contact with healthcare providers as well as mental health services. To address this, we performed a sensitivity analysis by excluding neurodevelopmental disorders diagnosed within 1 year after diabetes diagnosis. The magnitude of risk estimates remained similar although with some loss of statistical power. Furthermore, we cannot rule out reverse causation between neurodevelopmental disorders and glycaemic control. Since the manifestations of neurodevelopmental disorders change with age and over time [34], patients with type 1 diabetes with undiagnosed neurodevelopmental disorders may have inferior neuropsychological functions that can negatively affect their abilities to manage their diabetes, resulting in poor glycaemic control.

This is the first large nationwide register-based study with prospectively collected data specifically investigating the association of childhood-onset type 1 diabetes and neurodevelopmental disorders, while determining the role of glycaemic control in this association. The selection of matched reference individuals from the general population and the exact matching allowed us to control for possible confounding from sex, birth year and birth county. The study took place in Sweden, a country with a tax-funded healthcare system with universal access and free-of-charge paediatric care, which reduced possible confounding from socioeconomic factors [35]. This is important since earlier research indicated a link between low socioeconomic status and both poor glycaemic control [36] and neurodevelopmental disorders [37]. In our study, we adjusted for socioeconomic status, using parental education as a proxy.

Our study also has some limitations. Reverse causation between neurodevelopmental disorders and glycaemic control cannot be fully ruled out in an observational study. Moreover, because of the registration-based nature of the study, we could not monitor the glycaemic control of every patient precisely from diabetes diagnosis. To address this concern, we repeated the analysis in patients with first HbA_1c_ documented within the first 3 months after diagnosis, as HbA_1c_ primarily reflects 3-month average glycaemic control [38], and observed similar associations. Additionally, because of the lack of data, we were not able to control for prenatal exposure to maternal gestational diabetes, which is a potential risk factor for neurodevelopmental disorders in offspring [39]. Despite our large sample size of individuals with childhood-onset type 1, we had limited statistical power to examine co-occurrence of neurodevelopmental disorders and levels of intellectual disability as outcomes. Among patients with diabetes diagnosed with subsequent neurodevelopmental disorders, only 22 (5%) received two or more diagnoses on the same date, and this corresponded to 201 (6%) among matched reference individuals. In total, 40 patients with diabetes were diagnosed with intellectual disability, among whom 34 (85%) were mild and 6 (15%) were moderate to severe. This was similar to levels of intellectual disability diagnosed in the matched reference individuals (271 [88%] mild and 36 [12%] moderate to severe). Also, we do not have data on other neurodevelopmental disorders such as communication or specific learning disorders, and we had limited statistical power to evaluate the association between exposure to severe hypoglycaemia or diabetic ketoacidosis and later risk of neurodevelopmental disorders, which have been previously associated with intellectual disability and ADHD. Besides, HbA_1c_ values were not available for the matched reference individuals. Given the relatively young age of the study sample, prediabetes and undiagnosed type 2 diabetes, and thus the HbA_1c_ levels of the matched reference individuals, were presumed to be close to normal. Although we cannot fully exclude the possibility of a bias towards underestimation of risk due to disturbed glucose metabolism from other reasons in the matched reference individuals, we were similarly unable to evaluate the optimal HbA_1c_ target regarding future risk of neurodevelopmental disorders. We used the current cut-off to be consistent with a previous Danish study [18], but a similar pattern of association was observed when glycaemic control was categorised differently (ESM Table 8 and 9).

Several implications for clinicians may be derived from the present study. First, our findings further support existing evidence that individuals with childhood-onset type 1 diabetes are at higher risk of neurodevelopmental disorders. Second, we demonstrated that glycaemic control is an independent risk factor for clinically diagnosed neurodevelopmental disorders. Thus, optimal diabetes management along with psychological care is crucial for children and adolescents with type 1 diabetes. Paediatricians should be aware of the relationship between treatment adherence in individuals with type 1 diabetes and impairment in executive functions. Neurocognitive assessment for neurodevelopmental disorders should be offered to children and adolescents with self-management difficulties, suboptimal glycaemic control, and unexplained academic problems. Appropriate educational adjustment, and social and family support, should be available to individuals with childhood-onset type 1 diabetes and neurodevelopmental disorders.

Future longitudinal studies, with information on a wider range of diabetes-related factors such as episodes and severity of acute complications, are warranted in order to gain more insights into the aetiology of neurodevelopmental disorders in childhood-onset type 1 diabetes. Research on strategies that can integrate paediatric psychological services and diabetes care are needed for prevention and early detection of the comorbidity.

## Supplementary information

ESM (PDF 260 kb)

## Data Availability

Data from the Swedish registers that used in this study can be requested from the National Board of Health Welfare in Sweden (www.socialstyrelsen.se). According to Swedish legislation, the authors are not allowed to distribute or make individual level data directly available to other researchers under our current approval of using these registers. Detailed information regarding register linkage can be obtained from the Swedish National Board of Health and Welfare (https://www.socialstyrelsen.se/en/statistics-and-data/registers/).

## Abbreviations

ADHD: Attention-deficit/hyperactivity disorder
ASD: Autism spectrum disorders
ISPAD: International Society for Pediatric and Adolescent Diabetes
