# Supplementary material for: Poor glycaemic control is associated with increased risk of neurodevelopmental disorders in childhood-onset type 1 diabetes: a population-based cohort study
Source: Diabetologia. 2021 Jan 16;64(4):767–77. doi: 10.1007/s00125-020-05372-5 (PMC7940269; doi:10.1007/s00125-020-05372-5)
Supplement: Supplementary file 1 — (PDF 260 kb) [file 125_2020_5372_MOESM1_ESM.pdf]

ESM Table 1. Descriptions of used Swedish registers.

Register, coverage period	Summary of available information
National Diabetes Register,1996-	The Swedish Diabetes Registry was started from 1996 and it defines type 1 diabetes as receiving insulin treatment and diagnosis before or at age 30, which has been validated in 97% of cases (1).
SWEDIABKIDS Database, 2000-	The Swediabkids database was established in 2000 and collected information for more than 95% of Swedish children and adolescents with type 1 diabetes, which contains outpatient records from all paediatric diabetes centres in Sweden. Since 2007 the registry has included data on almost all (98%) children and adolescents with diabetes in Sweden, and since 2008 information on severe hypoglycaemia is recorded (2).
National Patient Register (NPR), 1964-	Data on inpatient (nationwide coverage since 1987) and outpatient specialist health care facilities (since 2001). Diagnoses, coded using the International Classification of Diseases (ICD 7-10), and are given by a consultant physician at the time of discharge. The validity of many diagnoses has been evaluated and found to be 95-100% (3).
Clinical Database for Child and Adolescent Psychiatry in Stockholm (PASTILL), 2001-	Data from child and adolescent psychiatric in– and outpatient care within Stockholm County with 96% validity proved for neurodevelopmental disorders (4).
Habilitation Register (HAB), 1997-	Data on use of Stockholm County Habilitation Services due to disability (intellectual disability, autism spectrum disorders, motor, vision and hearing impairment) (4).
The Halmstad University Register on Pupils with Intellectual Disability (HURPID), 2001-	School education is compulsory for all children between the ages of 7 and 16 residing in Sweden; children with intellectual disability who are not be able to fulfil the learning goals in the ordinary school are entitled to a special type of school that is physically integrated with the regular educational system. The data from HURPID comes from such upper secondary schools for pupils with intellectual disability (5).
Multi-Generation Register (MGR), 1932-	Swedish residents linked to their parents. Enables identification of relatives and construction of extended family structure.
Total Population Register, 1961-	Marital status, Citizenships, immigration and emigration (6).
Cause of Death Register, 1952-	Primary and contributory causes of death and date of death (7).
Migration Register, 1961-	Information on the date of emigration events (6).
Education Register, 1985-	Established in 1985, consists of data from educational institutions in Sweden updated once a year. 31 Earlier registers of the population's level of education had been produced in the 1960 and 1970 censuses (8).
Integrated Database for Labor and Market Research (LISA), 1990-	Annual information on education level, personal and family income, unemployment status, and

	social welfare benefits since 1990 for all individuals from 16 years of age (9).

ESM Table 2. ICD codes used to define psychiatric disorders in the patients and their parents.

	ICD-8	ICD-9	ICD-10
	(1969-1986)	(1987-1996)	(1997-)
**Diagnoses of study outcomes**			
**Primary outcomes:**			
Any Neurodevelopmental Disorders	Any code from specific categories of psychiatric disorders	Any code from specific categories of psychiatric disorders	Any code from specific categories of psychiatric disorders
**Secondary outcomes, specific categories of neurodevelopmental disorders**			
Attention deficit/hyperactivity disorders	-	314	F90
Autism spectrum disorder	-	299	F84
Intellectual disability	310-315	317-319	F70-F79
**Psychiatric diagnoses for adjustment**			
**Any psychiatric disorders**	Any code from other psychiatric disorders plus any neurodevelopmental disorders	Any code from other psychiatric disorders plus any neurodevelopmental disorders	Any code from other psychiatric disorders plus any neurodevelopmental disorders
**Other psychiatric disorders**	Any code from specific categories of psychiatric disorders	Any code from specific categories of psychiatric disorders	Any code from specific categories of psychiatric disorders
**Specific categories of psychiatric disorders:**			
Psychotic disorders	295, 297-299	295, 297, 298	F20-F29
Mood disorders	296, 300.40	296, 300E, 311	F30-F39
Anxiety disorders	300.00-300.30,300.50-300.99, 307	300, 300A-300D, 300F-300X, 308-309	F40-F45, F48
Eating disorders	-	307B, 307F	F50
Substance misuse	291, 303, 304	291, 303, 304, 305A, 305X	F10-F19

Behavioral disorders	-	-	F91
Personality disorders	301	301	F60-F62, F69

ICD: International Statistical Classification of Disease and Related Health Problems.

ESM Table 3. Hazard ratios (HR) with 95% confidence intervals (95%CI) for neurodevelopmental disorders among patients with childhood-onset type 1 diabetes diagnosed after 2008, according to time-varying HbA_{1c} levels.

Variable	Time-varying HbA_{1c} levels			HbA_{1c} 1% (10 mmol/mol) change
	<7.5% (<58 mmol/mol)	7.5,8.6% (58,70 mmol/mol)	>8.6% (>70 mmol/mol)	
Any neurodevelopmental disorders				
Model 1[a]	1 (reference)	1.44 (0.94,2.18)	4.22 (2.78,6.42)	1.45 (1.31,1.61)
Model 2[b]	1 (reference)	1.64 (1.03,2.61)	4.77 (2.89,7.67)	1.55 (1.38, 1.74)
Model 3[c]	1 (reference)	1.46 (0.90,2.36)	4.13 (2.59,6.59)	1.51 (1.35,1.69)
ADHD				
Model 1	1 (reference)	1.46 (0.90,2.37)	4.18 (2.59,6.77)	1.45 (1.30,1.61)
Model 2	1 (reference)	1.64 (0.95, 2.83)	4.98 (2.85,8.70)	1.60 (1.43,1.80)
Model 3	1 (reference)	1.37 (0.78,2.40)	3.97 (2.31,6.83)	1.54 (1.35,1.75)
ASD				
Model 1	1 (reference)	1.63 (0.69,3.84)	2.17 (0.76,6.18)	1.18 (0.90,1.54)
Model 2	1 (reference)	1.87 (0.72,4.84)	2.72 (0.79,9.41)	1.21 (0.88,1.67)
Model 3	1 (reference)	2.22 (0.87,5.67)	2.64 (0.89,7.82)	1.25 (0.92,1.70)
Intellectual disability				
Model 1	1 (reference)	0.47 (0.06,3.91)	11.74 (3.16,43.62)	2.66 (1.74,4.06)
Model 2	1 (reference)	0.86 (0.11,6.67)	13.28 (1.89,93.19)	2.96 (1.69,5.18)
Model 3	1 (reference)	0.62 (0.07,5.45)	19.73 (3.83,101.5)	2.82 (1.78,4.46)

[a] Model 1: stratified Cox regression on age at type 1 diabetes diagnosis and birth year, adjusted for time-updated diabetes duration.
[b] Model 2: additionally adjusted for sex, psychiatric morbidity prior to inclusion, parental psychiatric history and highest parental education level.
[c] Model 3: additionally adjusted for time-updated episodes of severe hypoglycaemia and diabetic ketoacidosis.

ESM Table 4. Incidence rate and hazard ratios (HR) with 95% confidence intervals (95%CI) for neurodevelopmental disorders diagnosed after 1 year of follow-up among patients with childhood-onset type 1 diabetes and matched reference individuals from general population, according to mean HbA_{1c} levels.

Variable	Matched reference individuals	Overall type 1 diabetes	Type 1 diabetes grouped by mean HbA_{1c} during follow-up		
			<7.5% (<58 mmol/mol)	7.5,8.6% (58,70 mmol/mol)	>8.6% (>70 mmol/mol)
**Any neurodevelopmental disorders**					
**Number of outcomes /person-years**	3,060/500,599	385/50,050	115/16,888	179/24,487	91/8,676
**Incidence rate/per 1000 person-years[a]**	6.11	7.69	6.81	7.31	10.49
**Crude model[b]**	1 (reference)	1.25 (1.13,1.39)	1.04 (0.86,1.26)	1.19 (1.02,1.39)	1.95 (1.56,2.45)
**Adjusted model[c]**	1 (reference)	1.27 (1.14,1.41)	1.11 (0.91,1.35)	1.21 (1.03,1.42)	1.75 (1.39,2.21)
**ADHD**					
**Number of outcomes /person-years**	2,309/500,599	289/50,050	75/16,888	141/24,487	73/8,676
**Incidence rate/per 1000 person-years**	4.61	5.77	4.44	5.75	8.41
**Crude model**	1 (reference)	1.25 (1.10,1.41)	0.90 (0.71,1.14)	1.24 (1.04,1.48)	2.11 (1.64,2.72)
**Adjusted model**	1 (reference)	1.25 (1.11,1.42)	0.96 (0.76,1.22)	1.26 (1.05,1.50)	1.84 (1.42,2.39)
**ASD**					
**Number of outcomes /person-years**	652/500,599	81/50,050	37/16,888	32/24,487	12/8,676
**Incidence rate/per 1000 person-years**	1.30	1.62	2.19	1.31	1.38
**Crude model**	1 (reference)	1.23 (0.98,1.56)	1.38 (0.98,1.94)	1.07 (0.74,1.54)	1.36 (0.74,2.49)
**Adjusted model**	1 (reference)	1.26 (1.00,1.59)	1.45 (1.03,2.06)	1.07 (0.74,1.54)	1.36 (0.74,2.52)
**Intellectual disability**					
**Number of outcomes /person-years**	307/500,599	38/50,050	10/16,888	19/24,487	9/8,676
**Incidence rate/per 1000 person-years**	0.61	0.76	0.59	0.78	1.04
**Crude model**	1 (reference)	1.22 (0.87,1.71)	1.02 (0.53,1.95)	1.20 (0.75,1.94)	1.66 (0.82,3.36)

**Adjusted model**	1 (reference)	1.28 (0.90,1.81)	1.12 (0.58,2.18)	1.31 (0.80,2.14)	1.42 (0.69,2.95)

[a] Number of outcomes per 1000 person-years.

[b] Crude Model: stratified Cox regression, each matched group as stratum.

[c] Adjusted Model: additionally adjusted for psychiatric morbidity prior to inclusion, parental psychiatric history and highest parental education level.

ESM Table 5. Hazard ratios (HR) with 95% confidence intervals (95%CI) for neurodevelopmental disorders diagnosed after 1 year of follow-up among patients with childhood-onset type 1 diabetes, according to time-varying HbA_{1c} levels.

Variable	Time-varying HbA_{1c} levels			HbA_{1c} 1% (10 mmol/mol) change
	<7.5% (<58 mmol/mol)	7.5,8.6% (58,70 mmol/mol)	>8.6% (>70 mmol/mol)	
Any neurodevelopmental disorders				
Model 1[a]	1 (reference)	1.56 (1.18,2.06)	3.64 (2.76,4.81)	1.37 (1.29,1.46)
Model 2[b]	1 (reference)	1.62 (1.20,2.19)	3.70 (2.74,5.00)	1.38 (1.29,1.47)
ADHD				
Model 1	1 (reference)	1.65 (1.19,2.29)	4.18 (3.02,5.78)	1.44 (1.34,1.53)
Model 2	1 (reference)	1.68 (1.19,2.42)	4.14 (2.90,5.90)	1.44 (1.35,1.55)
ASD				
Model 1	1 (reference)	1.43 (0.82,2.51)	2.40 (1.35,4.26)	1.10 (0.93,1.29)
Model 2	1 (reference)	1.57 (0.86,2.90)	2.84 (1.52,5.28)	1.14 (0.97,1.35)
Intellectual disability				
Model 1	1 (reference)	1.59 (0.68,3.73)	3.44 (1.44,8.24)	1.35 (1.07,1.70)
Model 2	1 (reference)	1.95 (0.67,5.64)	3.93 (1.38,11.22)	1.41 (1.11,1.79)

[a] Model 1: stratified Cox regression on age at type 1 diabetes diagnosis and birth year, adjusted for time-updated diabetes duration.

[b] Model 2: additionally adjusted for sex, psychiatric morbidity prior to inclusion, parental psychiatric history and highest parental education level.

ESM Table 6. Incidence rate and hazard ratios (HR) with 95% confidence intervals (95%CI) for neurodevelopmental disorders among patients with childhood-onset type 1 diabetes (first HbA_{1c} documented within 3 month after diagnosis) compared with matched reference individuals from general population, according to mean HbA_{1c} levels.

Variable	Matched reference individuals	Overall type 1 diabetes	Type 1 diabetes grouped by mean HbA_{1c} during follow-up		
			**<7.5% (<58 mmol/mol)**	**7.5,8.6% (58,70 mmol/mol)**	**>8.6% (>70 mmol/mol)**
**Any neurodevelopmental disorders**					
**Number of outcomes /person-years**	2,278/364,303	288/36,392	94/12,930	133/17,651	61/5,811
**Incidence rate/per 1000 person-years**[a]	6.25	7.91	7.27	7.54	10.50
**Crude model**[b]	1 (reference)	1.26 (1.12,1.43)	1.08 (0.87,1.33)	1.21 (1.01,1.45)	1.96 (1.49,2.58)
**Adjusted model**[c]	1 (reference)	1.29 (1.14,1.46)	1.16 (0.93,1.44)	1.25 (1.04,1.50)	1.75 (1.32,2.33)
**ADHD**					
**Number of outcomes /person-years**	1,694/364,303	219/36,392	62/12,930	105/17,651	52/5,811
**Incidence rate/per 1000 person-years**	4.65	6.02	4.80	5.95	9.95
**Crude model**	1 (reference)	1.29 (1.12,1.49)	0.95 (0.73,1.24)	1.29 (1.05,1.58)	2.29 (1.70,3.10)
**Adjusted model**	1 (reference)	1.32 (1.14,1.53)	1.02 (0.78,1.33)	1.34 (1.09,1.65)	2.00 (1.46,2.73)
**ASD**					
**Number of outcomes /person-years**	510/364,303	62/36,392	30/12,930	24/17,651	8/5,811
**Incidence rate/per 1000 person-years**	1.40	1.70	2.32	1.36	1.89
**Crude model**	1 (reference)	1.21 (0.93,1.58)	1.38 (0.94,2.02)	1.04 (0.68,1.58)	1.26 (0.60,2.64)
**Adjusted model**	1 (reference)	1.24 (0.95,1.62)	1.47 (0.99,2.16)	1.02 (0.67,1.57)	1.28 (0.60,2.71)
**Intellectual disability**					
**Number of outcomes /person-years**	230/364,303	24/36,392	7/12,930	13/17,651	4/5,811
**Incidence rate/per 1000 person-years**	0.63	0.66	0.54	0.74	0.69
**Crude model**	1 (reference)	1.04 (0.68,1.58)	0.94 (0.43,2.04)	1.09 (0.61,1.93)	1.06 (0.38,2.98)

**Adjusted model**	1 (reference)	1.06 (0.69,1.63)	1.00 (0.45,2.20)	1.16 (0.64,2.09)	0.91 (0.32,2.59)

[a] Number of outcomes per 1000 person-years.

[b] Crude Model: stratified Cox regression, each matched group as stratum, adjusted for time-updated diabetes duration.

[c] Adjusted Model: additionally adjusted for psychiatric morbidity prior to inclusion, parental psychiatric history and highest parental education level.

ESM Table 7. Hazard ratios (HR) with 95% confidence intervals (95%CI) for Neurodevelopmental Disorders among Patients with Childhood-onset Type 1 Diabetes (first HbA_{1c} documented within 3 month after diagnosis), according to time-varying HbA_{1c} levels.

Variable	Time-varying HbA_{1c} levels			HbA_{1c} 1% (10 mmol/mol) change
	<7.5% (<58 mmol/mol)	7.5,8.6% (58,70 mmol/mol)	>8.6% (>70 mmol/mol)	
Any neurodevelopmental disorders				
Model 1[a]	1 (reference)	1.54 (1.13,2.09)	3.29 (2.40,4.51)	1.33 (1.24,1.44)
Model 2[b]	1 (reference)	1.67 (1.20,2.33)	3.32 (2.33,4.74)	1.34 (1.23,1.46)
ADHD				
Model 1	1 (reference)	1.52 (1.06,2.19)	3.75 (2.60,5.40)	1.40 (1.30,1.51)
Model 2	1 (reference)	1.57 (1.06,2.32)	3.58 (2.37,5.40)	1.40 (1.28,1.54)
ASD				
Model 1	1 (reference)	1.43 (0.80,2.55)	2.12 (1.09,4.12)	1.07 (0.88,1.30)
Model 2	1 (reference)	1.72 (0.89,3.33)	2.69 (1.26,5.71)	1.10 (0.90,1.36)
Intellectual disability				
Model 1	1 (reference)	2.30 (0.88,6.05)	2.70 (0.87,8.32)	1.28 (0.90,1.83)
Model 2	1 (reference)	4.50 (1.09,18.61)	3.39 (0.78,14.76)	1.27 (0.85,1.88)

[a] Model 1: stratified Cox regression on age at type 1 diabetes diagnosis and birth year, adjusted for time-updated diabetes duration.

[b] Model 2: additionally adjusted for sex, psychiatric morbidity prior to inclusion, parental psychiatric history and highest parental education level.

ESM Table 8. Incidence rate and hazard ratios (HRs) with 95% confidence interval (CI) for neurodevelopmental disorders among patients with childhood-onset type 1 diabetes compared with matched reference individuals from the general population, according to mean HbA_{1c} levels categorized according to International Society for Paediatric and Adolescent Diabetes cut-offs.

Variable	Matched reference individuals	Overall type 1 diabetes	Type 1 diabetes grouped by mean HbA_{1c} during follow-up		
			<7.5% (<58 mmol/mol)	7.5,8.6% (58,70 mmol/mol)	>8.6% (>70 mmol/mol)
**Any neurodevelopmental disorders**					
**Number of outcomes /person-years**	3,066/501,314	398/50,051	59/7,912	241/33,463	98/8,675
**Incidence rate/per 1000 person-years[a]**	6.12	7.95	7.46	7.20	11.30
**Crude model[b]**	1 (reference)	1.29 (1.17,1.44)	1.08 (0.83,1.41)	1.17 (1.02,1.34)	2.10 (1.69,2.62)
**Adjusted model[c]**	1 (reference)	1.31 (1.18,1.46)	1.14 (0.87,1.50)	1.21 (1.05,1.38)	1.90 (1.51,2.37)
**ADHD**					
**Number of outcomes /person-years**	2,313/501,314	297/50,051	39/7,912	180/33,463	78/8,676
**Incidence rate/per 1000 person-years**	4.61	5.93	4.93	5.38	8.99
**Crude model**	1 (reference)	1.28 (1.14,1.45)	0.97 (0.69,1.34)	1.15 (0.98,1.34)	2.25 (1.76,2.88)
**Adjusted model**	1 (reference)	1.29 (1.14,1.46)	1.02 (0.73,1.42)	1.18 (1.01,1.38)	1.98 (1.53,2.55)
**ASD**					
**Number of outcomes /person-years**	654/501,314	84/50,051	20/7,912	51/33,463	13/8,676
**Incidence rate/per 1000 person-years**	1.30	1.68	2.53	1.52	1.50
**Crude model**	1 (reference)	1.28 (1.02,1.61)	1.49 (0.93,2.39)	1.18 (0.88,1.57)	1.47 (0.82,2.64)
**Adjusted model**	1 (reference)	1.31 (1.04,1.65)	1.57 (0.97,2.52)	1.20 (0.89,1.61)	1.48 (0.82,2.68)
**Intellectual disability**					
**Number of outcomes /person-years**	307/501,314	40/50,051	4/7,912	26/33,463	10/8,675
**Incidence rate/per 1000 person-years**	0.61	0.80	0.51	0.78	1.15
**Crude model**	1 (reference)	1.29 (0.93,1.79)	0.77 (0.28,2.14)	1.27 (0.84,1.91)	1.84 (0.94,6.63)

**Adjusted model**	1 (reference)	1.35 (0.96,1.90)	0.75 (0.27,2.10)	1.45 (0.95,2.20)	1.61 (0.80,3.23)

[a] Number of outcomes per 1000 person-years.

[b] Crude Model: stratified Cox regression, each matched group as stratum, adjusted for time-updated diabetes duration.

[c] Adjusted Model: additionally adjusted for psychiatric morbidity prior to inclusion, parental psychiatric history and highest parental education level.

ESM Table 9. Hazard ratios (HR) with 95% confidence intervals (95%CI) for neurodevelopmental disorders among patients with childhood-onset type 1 diabetes, according to time-varying HbA_{1c} levels categorized according to International Society for Paediatric and Adolescent Diabetes cut-offs.

Variable	Time-varying HbA_{1c} levels			HbA_{1c} 1% (10 mmol/mol) change
	<7.5% (<58 mmol/mol)	7.5,8.6% (58,70 mmol/mol)	>8.6% (>70 mmol/mol)	
Any neurodevelopmental disorders				
Model 1[a]	1 (reference)	1.23 (0.91,1.67)	3.34 (2.44,4.57)	1.37 (1.30,1.46)
Model 2[b]	1 (reference)	1.30 (0.92,1.84)	3.36 (2.35,4.80)	1.37 (1.30,1.46)
ADHD				
Model 1	1 (reference)	1.32 (0.92,1.90)	3.89 (2.70,5.63)	1.43 (1.34,1.53)
Model 2	1 (reference)	1.37 (0.91,2.07)	3.81 (2.50,5.81)	1.45 (1.35,1.55)
ASD				
Model 1	1 (reference)	0.76 (0.41,1.38)	1.60 (0.87,2.96)	1.11 (0.95,1.30)
Model 2	1 (reference)	0.82 (0.42,1.61)	1.84 (0.93,3.63)	1.14 (0.97,1.35)
Intellectual disability				
Model 1	1 (reference)	1.76 (0.76,4.07)	3.84 (1.61,9.18)	1.40 (1.12,1.75)
Model 2	1 (reference)	1.95 (0.67,5.65)	3.93 (1.38,11.22)	1.41 (1.11,1.79)

[a] Model 1: stratified Cox regression on age at type 1 diabetes diagnosis and birth year, adjusted for time-updated diabetes duration.

[b] Model 2: additionally adjusted for sex, psychiatric morbidity prior to inclusion, parental psychiatric history and highest parental education level.

References

1. Eeg,Olofsson K, Cederholm J, Nilsson PM, Zethelius B, Svensson AM, Gudbjornsdottir S, Eliasson B. Glycemic control and cardiovascular disease in 7,454 patients with type 1 diabetes: an observational study from the Swedish National Diabetes Register (NDR). Diabetes Care 2010;33:1640,1646
2. Samuelsson U, Akesson K, Peterson A, Hanas R, Hanberger L. Continued improvement of metabolic control in Swedish pediatric diabetes care. Pediatr Diabetes 2018;19:150,157
3. Ludvigsson JF, Andersson E, Ekbom A, Feychting M, Kim J,L, Reuterwall C, Heurgren M, Olausson PO. External review and validation of the Swedish national inpatient register. BMC Public Health 2011;11:450
4. Idring S, Rai D, Dal H, Dalman C, Sturm H, Zander E, Lee BK, Serlachius E, Magnusson C. Autism spectrum disorders in the Stockholm Youth Cohort: design, prevalence and validity. PloS one 2012;7:e41280
5. Lundh A, Forsman M, Serlachius E, Lichtenstein P, Landen M. Outcomes of child psychiatric treatment. Acta Psychiatr Scand 2013;128:34,44
6. Ludvigsson JF, Almqvist C, Bonamy A,KE, Ljung R, Michaëlsson K, Neovius M, Stephansson O, Ye W. Registers of the Swedish total population and their use in medical research. Eur J Epidemiol 2016;31:125,136
7. Brooke HL, Talbäck M, Hörnblad J, Johansson LA, Ludvigsson JF, Druid H, Feychting M, Ljung R. The Swedish cause of death register. Eur J Epidemiol 2017;32:765,773
8. Sweden S. *Educational attainment of the population 2010*. Statistics Sweden: Stockholm 2011;
9. Ludvigsson JF, Svedberg P, Olén O, Bruze G, Neovius M. The longitudinal integrated database for health insurance and labour market studies (LISA) and its use in medical research. Eur J Epidemiol 2019;34:423,437
